# Boosting *Solanum tuberosum* resistance to *Alternaria solani* through green synthesized ferric oxide (Fe_2_O_3_) nanoparticles

**DOI:** 10.1038/s41598-024-52704-1

**Published:** 2024-01-29

**Authors:** Sadaf Anwaar, Dur-e-Shahwar Ijaz, Tauseef Anwar, Huma Qureshi, Moona Nazish, Abdulwahed Fahad Alrefaei, Mikhlid H. Almutairi, Sultan N. Alharbi

**Affiliations:** 1 Department of Biological Sciences, International Islamic University, Islamabad, 44000 Pakistan; 2 Department of Botany, The Islamia University of Bahawalpur, Bahawalpur, 63100 Pakistan; 3 Department of Botany, University of Chakwal, Chakwal, 48800 Pakistan; 4 Department of Botany, Rawalpindi Women University, Rawalpindi, 46300 Pakistan; 5 Department of Zoology, College of Science, King Saud University, Riyadh, 11451 Saudi Arabia; 6 Department of Surgery and Cancer, Imperial College London, Hammersmith Campus, London, UK

**Keywords:** Microbiology, Plant sciences

## Abstract

Potato (*Solanum tuberosum*) is the third crucial global crop facing threats from *Alternaria solani*, a necrotrophic fungal pathogen causing early blight disease. Beyond crop impact, it leads to substantial production reduction and economic losses worldwide. This study introduces a green synthesis method for producing Ferric Oxide nanoparticles (FNPs) using dried Guava (*Psidium guajava*) leaves. Guava leaf extract acts as a reducing agent, with iron (III) chloride hexahydrate (FeCl_3_·6H_2_O) as the oxidizing agent. This study employed various characterization techniques for Ferric Oxide nanoparticles (FNPs). Fourier Transform Infrared Spectroscopy (FTIR) revealed peaks at 877 cm^−1^, 1180 cm^−1^, 1630 cm^−1^, 1833 cm^−1^, 2344 cm^−1^, and 3614 cm^−1^, associated with Maghemite vibrations, polyphenol compounds, and amino acids. UV–Vis spectroscopy exhibited a characteristic absorbance peak at 252 nm for FNPs. Scanning Electron Microscope (SEM) images illustrated particle sizes of 29-41 nm, and Energy Dispersive Spectroscopy (EDS) indicated elemental composition. X-ray diffraction (XRD) confirmed crystalline FNPs with peaks at 26.78, 30.64, 36.06, 38.21, 43.64, 53.52, 57.42, 63.14 and 78.32. Disease resistance assays demonstrated FNPs’ effectiveness against *A. solani*, reducing disease incidence and severity. In the leaf detach assay, concentrations of 15, 10 and 5 mg/L showed a dose-dependent reduction in disease severity and incidence. The Greenhouse Assay confirmed FNPs’ concentration-dependent effect on disease incidence and severity. The study also explored FNPs’ potential as biocontrol agents showing no adverse effects on overall plant development. Additionally, the study highlighted the agronomic potential of FNPs in enhancing plant growth and development emphasizing their role as micronutrients in biofortification. The findings suggest the promising application of FNPs in plant protection and biofortification strategies.

## Introduction

The rapidly advancing field of nanotechnology is driving progress across various scientific disciplines. Nanoparticles, defined by at least one dimension with a diameter of under 100 nm, are present in clays, minerals, and bacterial byproducts. Positioned as the sixth revolutionary innovation, nanotechnology harnesses both the physical and chemical properties at the molecular level, offering applications ranging from medicine to agriculture. Coined by Richard Feynman in 1959, the term “nano” originates from the Greek word “nanos,” signifying one billionth of a meter^1^. Nanotechnology explores and manipulates matter at an incredibly small scale, yielding remarkable advances in various scientific disciplines^1^. With a market value projected to reach US$ 125 billion by 2024, nanotechnology revolutionizes industries like food, medicine, agriculture, cosmetics and environmental health^2^. Widely adopted in agriculture, nanotechnology employs various nanoparticles such as selenium, zinc oxide, titanium dioxide, iron oxide and silicon oxide known for their safe use in agriculture^3^.

Nano-biotechnology introduces tools like nanoparticles, nano-emulsions, nanowires and nano-capsules revolutionizing crop production. As the global population is projected to reach 10 billion by 2050, with increasing food demands, nanotechnology emerges as a promising solution to improve agricultural efficiency and address environmental challenges^4,5^. These nanomaterials facilitate the delivery of agrochemicals and macromolecules essential for plant growth, enhancing stress resistance and optimizing nutrient conditions^6^. Recognized as the sixth revolutionary innovation, nanotechnology employs physical and chemical properties at the molecular level spanning agriculture applications^7^. Within the agricultural sector introduction of nano fertilizers emerges as a boon overcoming the limitations of traditional fertilizers. Nano-fertilizers, categorized as macro-nanofertilizers, micro-nanofertilizers and nanoparticulate fertilizers offer sustainable solutions for enhancing nutrient use efficiency, reducing waste and promoting crop growth^8–10^. The efficient and environmentally friendly nature of nano and biofertilizers positions them as replacements for conventional chemical fertilizers, contributing to quick nutrient uptake and increased production^11^.

Among the various nanoparticles, iron oxide nanoparticles gain prominence due to their unique properties^12^. These nanoparticles exhibit superparamagnetic characteristics, low toxicity and microwave absorption capabilities making them versatile for applications in wastewater treatment, ferrofluids, catalysis, and biosensors^13,14^. The green synthesis of IONPs introduces an eco-friendly approach utilizing plant extracts rich in bioactive components as stabilizers, bio-reductants and capping agents. This green synthesis method aligns with the principles of green chemistry aiming to reduce waste and prevent environmental degradation^15^. Plant extracts from sources like pomegranate leaves and green tea leaves have demonstrated efficacy in the sustainable production of these nanoparticles^16^.

As the third-most significant food crop globally, *Solanum tuberosum* (potatoes) plays a crucial role in global food security. However, the prevalence of diseases, particularly early blight caused by *Alternaria solani* poses a significant threat to potato production. Early blight, characterized by black concentric ring-like lesions can result in severe yield losses if left untreated^17^. Belonging to the Myrtaceae family, *Psidium guajava*, commonly known as guava, has a significant historical legacy in traditional practices. Its versatile applications span antibacterial and anticancer properties, making different parts of the guava plant valuable in both culinary and folk medicinal contexts^18^. This study aims to enhance the natural immunity of *S. tuberosum* against the fungal pathogen *A. solani*. The objectives include the green synthesis and characterization of iron oxide nanoparticles utilizing them as potential nano-fungicides to boost plant immunity. The study further involves *in-vitro* and *in-vivo* evaluations of greenhouse-raised plants against *A. solani* shedding light on the promising role of nanotechnology in agriculture.

## Materials and methods

The research was conducted at the Applied Biotechnology and Genetic Engineering Laboratory in the Department of Biological Sciences, International Islamic University, Islamabad.

### Preparation of leaves extract

The freshly harvested leaves of *P. guajava* were washed with tap water, then dried for three weeks and finely powdered using an electric mortar. All procedures, from plant material collection to experimentation, strictly adhered to the guidelines and legislation of the Ethics Committee of the International Islamic University, Islamabad. To prepare the leaf extract, an exact quantity of 50 g of finely powdered leaves was measured with precision using an electric weighing balance. This powder was then mixed with 500 mL of sterilized double-distilled water in an Erlenmeyer flask, the volume was carefully measured with a graduated measuring cylinder. The flask was placed on a hot plate and heated to 50 °C, maintaining this temperature for a minimum of 30 min. Following this, the flask was covered with aluminum foil and transferred to a shaking incubator set at 37 °C for the next 24 h. The utilization of the shaking incubator aimed to achieve a phytochemically enriched extract. After a 24-h incubation period, the extract was retrieved and subjected to filtration. The filtration process involved passing it through a plain white muslin cloth first and subsequently through Whatman filter paper No.41, ensuring the attainment of a clear extract. This rigorous extraction method aimed to preserve the integrity and purity of the final extract for further analyses^19,20^.

### Green synthesis of ferric oxide nanoparticles (FNPs) and characterizations

**Figure 1:**
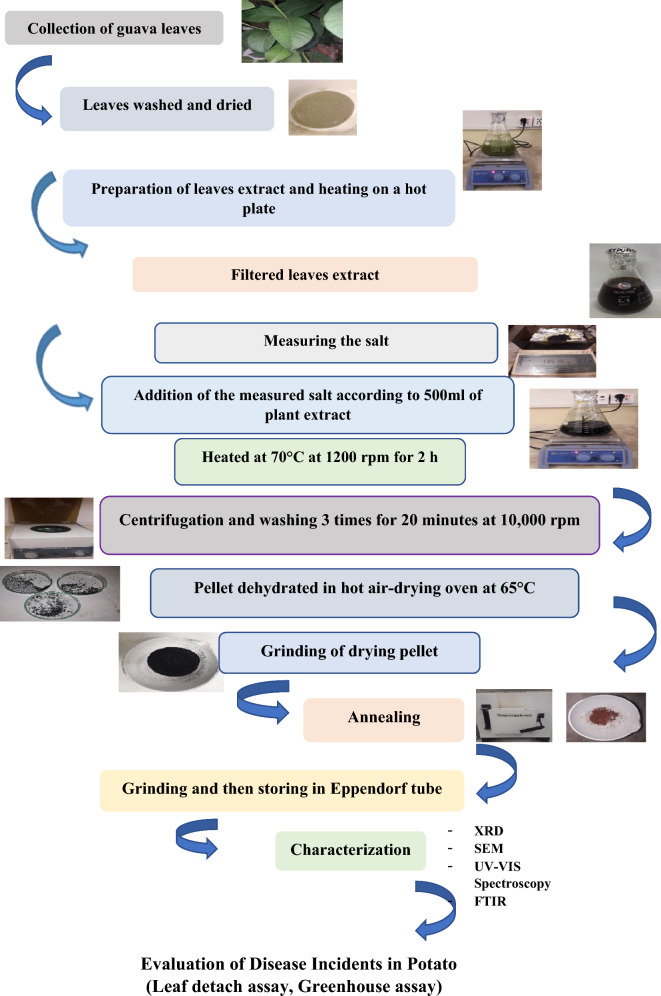
Schematic representation of Green synthesis of ferric oxide NPs and evaluation.

The synthesis involved the reaction of Iron (III) chloride hexahydrate (FeCl_3_·6H_2_O) with the guava leaf extract. The salt was measured (13.515 g) and added to the plant extract resulting in a color change indicating the production of FNPs. The mixture was stirred on a hot plate for two hours at 70 °C and 1200 rpm. The reaction mixture was cooled and centrifugation was carried out at 10,000 rpm for 15 min. The pellet obtained was washed with sterilized distilled water in successive steps and the washed pellet was finally transferred to a clean, sterilized petri dish. The petri dishes containing the pellet were subjected to oven drying until fully dehydrated. The dried pellets were then scraped off using a surgical blade. The completely dried nanoparticles were ground into a fine powder using a pestle and mortar and collected in a clean, dry China dish. The fine powder obtained was subjected to calcination in an electric muffle furnace at 450 °C for approximately 2 h. After cooling to room temperature, the calcinated nanoparticles were ground into a fine powder and stored in sterilized Eppendorf tubes^21–23^. Figure 1 illustrates a schematic representation of the protocol.

Characterization of synthesized FNPs involved X-ray diffraction (XRD), Fourier Transform Infrared Spectroscopy (FTIR), UV–Vis spectroscopy and Scanning Electron Microscope (SEM). The data was obtained from the Optics and Photonics Laboratory in the Physics Department at International Islamic University, Islamabad. The SEM images of the FNPs were provided by the Institute of Space Technology (IST), Islamabad.

### X-ray diffraction (XRD)

XRD was employed to analyze the crystalline structure of FNPs. This technique was instrumental in determining the phase purity and crystallographic form of the nanoparticles within the 2θ range, with θ spanning from 20° to 80°^24^.

### Fourier transform infrared spectroscopy (FTIR)

FTIR is a method that generates an infrared absorption spectrum, allowing the identification of chemical bonds in a molecule. The FTIR spectrum was obtained in the range of 400–4000 cm^−1^ and the examination of the dried powder of green-synthesized FNPs was also performed within this wavelength range^25^.

### Ultraviolet–visible spectroscopy (UV–vis spectroscopy)

UV–vis spectroscopy was employed to analyze the FNPs. The visible spectrum typically ranges from 400 to 800 nm while the ultraviolet region extends from 200 to 400 nm^26^.

### Scanning electron microscope (SEM) and energy dispersive spectroscopy (EDS)

SEM, EDS projects and scans a focused beam of electrons over a surface to generate images. This method allows for a detailed examination of the topography and composition of the surface.

## Constructing *Alternaria solani* inoculum

### Pure culture of *A. solani*

To establish a pure culture of *A. solani*, the sub-culturing technique was employed. Within the controlled laminar airflow, the culture plates containing *A. solani* culture were carefully handled. A loopful of the fungal culture was streaked onto a fresh, sterile Potato Dextrose Agar (PDA) plate. The plate was then sealed with parafilm and incubated for 48–72 h to monitor initial growth^27^. Following this, cultures were incubated at 25 °C for seven days in darkness to cultivate a pure fungal culture. Periodic checks were conducted to promptly identify and address any potential contaminations in the culture.

### Preparation of inoculum

A pristine cheesecloth was taken, folded in half and placed over a container. The incubated petri dish was carefully opened and 14 mL of double-distilled water (ddH_2_O) was gently poured into it. Using a glass microscope slide, the mycelium was scraped and poured over the cheesecloth. This process was repeated 2–3 times for thorough collection. An additional 14 mL of ddH_2_O was poured into the petri dish and any remaining mycelium was scraped off and poured again over the cheesecloth. The resultant conidial suspension was adjusted to a concentration of 106 spores/mL by diluting it with sterilized distilled water accompanied by the addition of Tween 20 as a surfactant^28^.

### Assessment of synthesized nanoparticles as nano-supplementation

The greenhouse experiments were initiated in October 2022. Potatoes were planted in medium-sized pots (depth 10 inches, diameter 30 cm). These pots contained fertile soils and were placed under controlled greenhouse conditions with temperatures ranging from 18 to 25 °C. Approximately 50 g potato tubers were individually sown in about 30 pots and these were provided with watering as needed depending on the plant’s growth. These pots were divided into different concentration groups and a control group. The green-synthesized FNPs were applied by immersing healthy potato tubers in various concentrations (5, 10, 15 mg/L) for 2 h. In contrast, potato tubers in the control group were soaked in water for the same duration. Subsequently, treated potato tubers were planted individually in plastic pots (one tuber per pot).

### Leaf detach assay

Fresh, fully developed and disease-free leaves were selectively gathered from both the treated and control groups. Compound leaves, each containing a minimum of three leaflets, were carefully selected from 5 to 8-week-old potato plants, with the exclusion of leaves displaying any indications of disease or infection^29,30^. Before inoculation with *Alternaria* fungal spore suspension on the abaxial surface, the leaflets were cleaned with sterile distilled water. A total of thirty fully grown leaflets, divided evenly between the treated and control groups (15 each) were placed in Petri dishes lined with water-soaked blotting paper. Three leaflets were placed in each dish, and two dishes were contained within each plastic box. On the abaxial side of each leaflet, a 20 μL drop of *A. solani* spore suspension was injected using a micropipette with a single point per leaflet. The plastic containers were incubated for seven days at a temperature of 25 °C and 90% relative humidity, maintaining a 12-h light cycle per day. Following the incubation period, symptoms were observed on the infected leaflets. After the seven-day incubation, disease severity was quantified using percentages to express the results.

The formulae applied for determining disease severity are as follows^31^:

$$Disease \;Severity (\%)=\frac{Sum \;of \;all \;disease \;rating \;(instances)}{Total \;no. \;of \;rating \times \;maximum \;disease \;grade}\times 100$$

$$Disease \;Incidence (\%)=\frac{No. \;of \;diseased \;leaves}{Total \;leaves}\times 100$$

The severity of the condition is evaluated using a rating scale ranging from 1 to 5. Rating 1 represents an absence of disease. Rating 2 indicates traces of disease, accounting for less than 1% of the affected tissue. Rating 3 signifies a light level of disease, covering 1–10% of the tissue. Rating 4 reflects a moderate degree of disease, involving 11–25% of the tissue. Rating 5 represents a severe condition, where more than 50% of the tissue is affected.

### Greenhouse assay

After 45 days both the treated and control potato plants underwent inoculation. This involved applying a 10 µL drop of *A. solani* suspension to the middle part of 15 randomly chosen leaflets strategically distributed across the lower, middle and upper regions. These leaflets were organized into sets of three with five sets in total of 9. To elevate humidity levels and enhance the infection rate, the plants were enclosed in plastic bags for approximately 24 h. Subsequently, the plants were allowed to continue growing in the greenhouse under optimal conditions at 26 °C^27^. After 10 days, the severity of the disease was calculated.

## Results and discussion

### Characterization techniques for Ferric Oxide nanoparticles (FNPs)

#### Fourier transform infrared spectroscopy (FTIR)

**Figure. 2:**
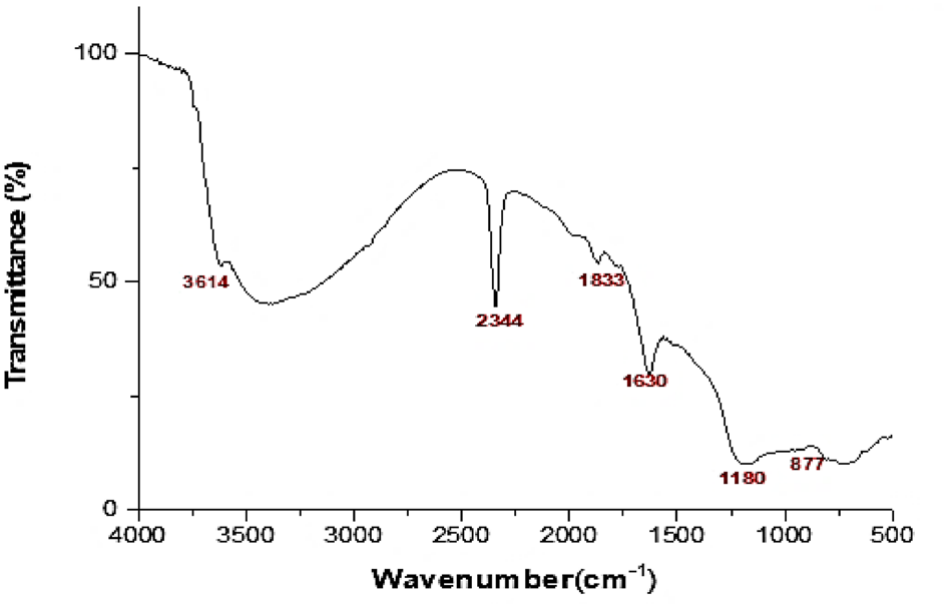
FTIR of Ferric Oxide nanoparticles.

The FTIR spectrum of FNPs showed visible peaks at 877 cm^−1^, 1180 cm^−1^, 1630 cm^−1^, 1833 cm^−1^, 2344 cm^−1^ and 3614 cm^−1^ (Fig. 2)^.^ The first peak observed at 877 cm^−1^ is due to Maghemite vibrations^32^. The C–O asymmetric stretching vibration is responsible for the peak at 1180 cm^−1^ in this spectrum. The absorption band at 1630 cm^−1^ which is related to C=O bond stretching, demonstrates the polyphenol compounds found in the plant extract and the amino acids that stabilized and served as a capping agent. Polyphenol compounds and phenyl groups are critical in the reduction of iron ions to FNPs. C=C=C stretching is responsible for the peak at 1833 cm^−1^^33^. According to the C–O vibrations assigned to the peak at 2344 cm^−1^, the presence of environmental CO_2_ can be connected to the water molecules present on the surface of the sample material at the peak at 2344 cm^−1^. The structural OH vibration is responsible for the peak at 3614 cm^−1^^34^.

### Ultraviolet-visible spectroscopy (UV–vis spectroscopy)

**Figure 3:**
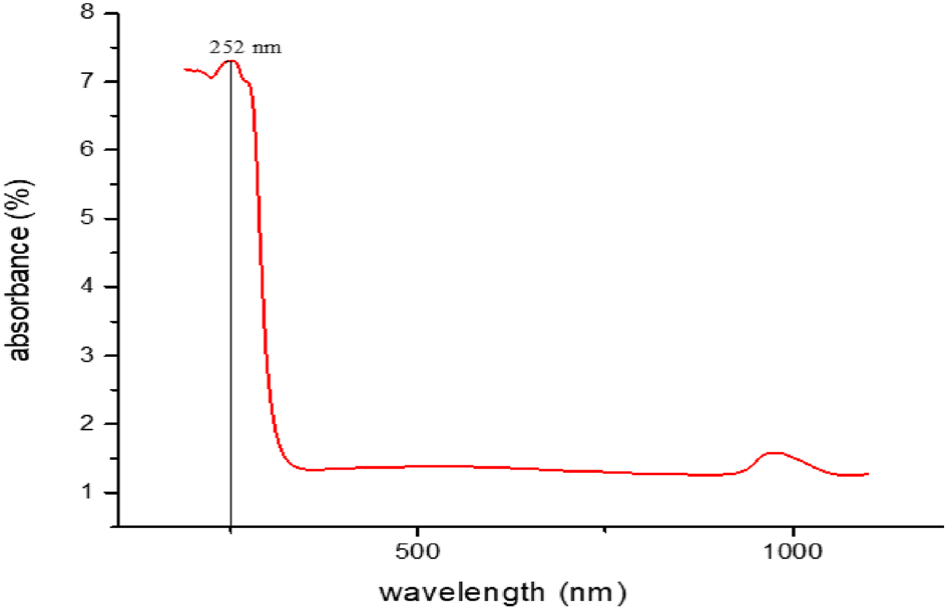
UV–Vis spectroscopy of Ferric Oxide nanoparticles.

The green synthesized FNPs showed a maximum absorbance range of 252 nm. The respective peak which showed maximum absorbance at 252 nm is a characteristic peak for FNPs (Fig. 3).

### Scanning electron microscope (SEM) and energy dispersive spectroscopy (EDS)

**Figure 4:**
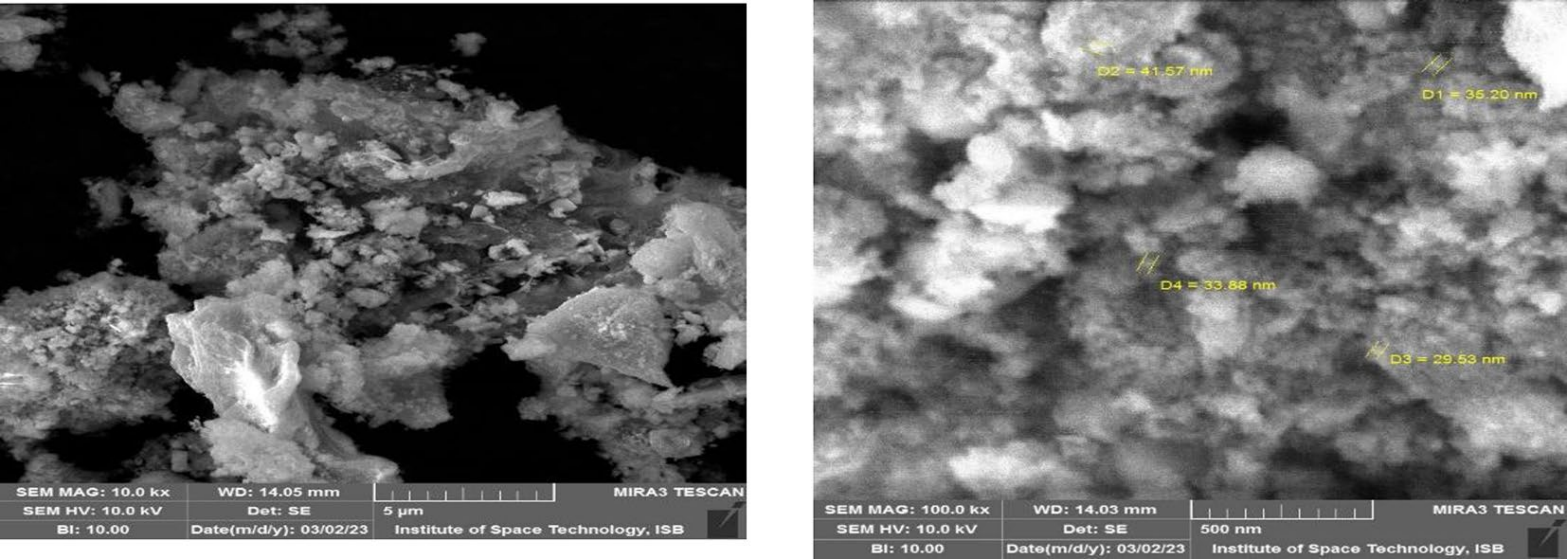
The SEM images obtained for the green synthesized Ferric Oxide nanoparticles from the guava leaves extract revealed particle sizes ranging between 29 and 41 nm.

**Figure 5:**
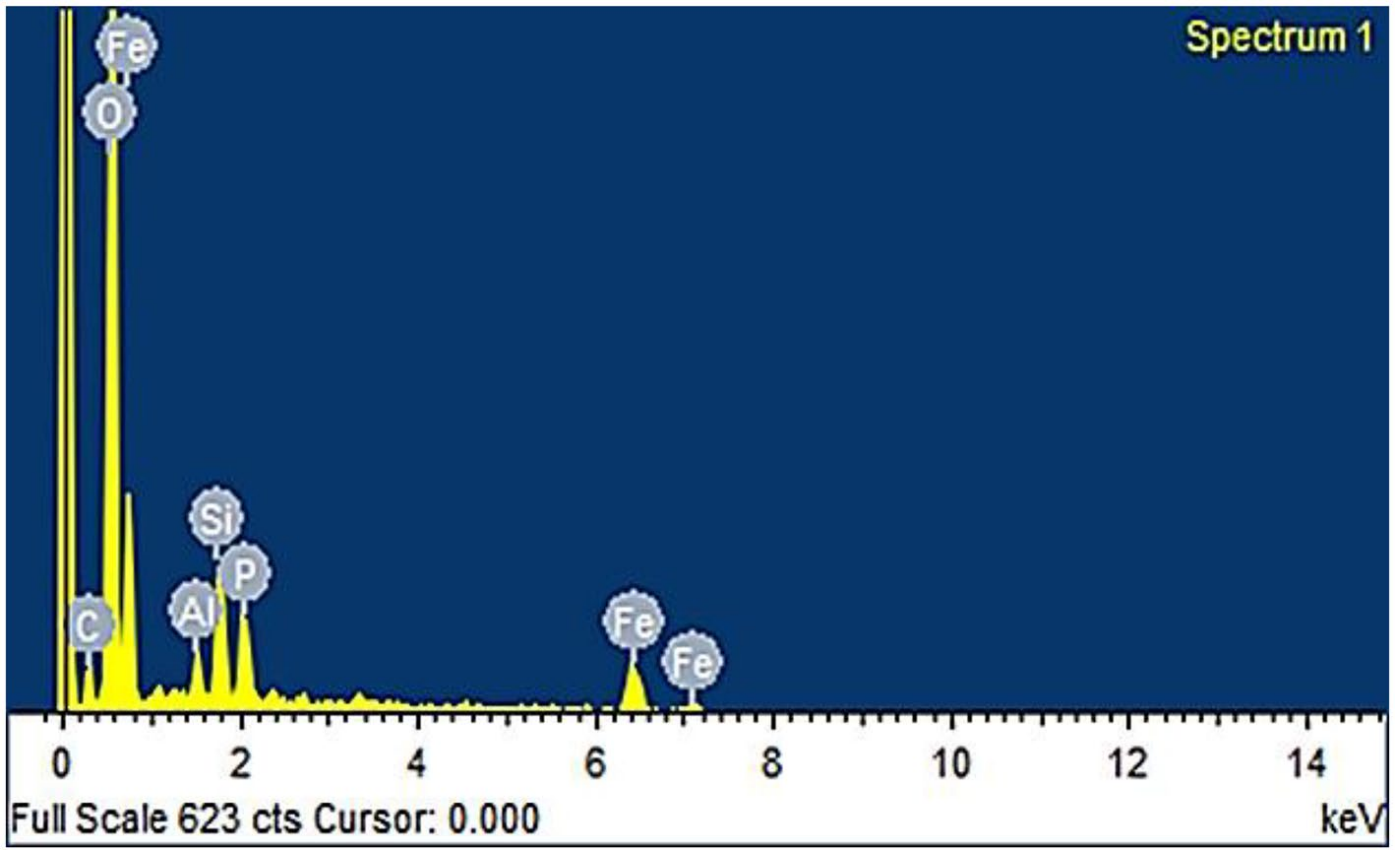
The results of the elemental analysis using Energy Dispersive Spectroscopy (EDS).

The SEM image obtained for the green synthesized FNPs from the guava leaves extract revealed particle sizes ranging between 29 and 41 nm (Fig. 4). The results of elemental analysis using Energy Dispersive Spectroscopy (EDS) which included the weight percentages of Fe, O, C, Al, and Si 48.44%, 35.56%, 4.24%, 1.22%, 5.73% (Fig. 5). The presence of O suggests that Ferric Oxide particles are produced when air and water react with the resulting FNPs, while the presence of Fe shows the emergence of iron-based particles. Given that these nanoparticles contain sizable amounts of carbon and oxygen, it is likely that organic groups containing C–O functional groups, such as flavonoids and polyphenol compounds, have been added to their surface^35^. According to EDS iron predominates a few silicon and aluminum residues. Iron is the main component of oxidized iron in steel and aluminum is important to the chemical composition of steel. Si and Al traces may originate from the chemical composition of the iron-carbon alloy (steel) or the powder used in continuous casting^32^.

### X-ray diffraction (XRD)

**Figure 6:**
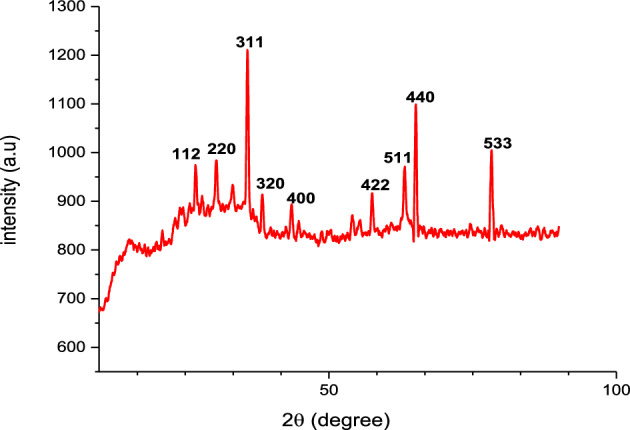
XRD graph of FNPs.

The XRD data of green synthesized FNPs showed defined peaks by Bragg reflection characteristics (Fig. 6). This data showed diffraction peaks at 2Ꝋ = (26.78), (30.64), (36.06), (38.21), (43.64), (53.52), (57.42), (63.14), (78.32) which were indexed as 112, 220, 311, 320, 400, 422, 511, 440 and 533 planes of FNPs. The Debye-Scherer equation is used to compute the average particle size (D) of the nanoparticle at peak 311. Analysis shows that the particles are spherical in shape and range in size from 72 nm^36^.

## Evaluation of FNPs’ potential use as nano-fungicides

### Leaf detach assay

**Figure 7:**
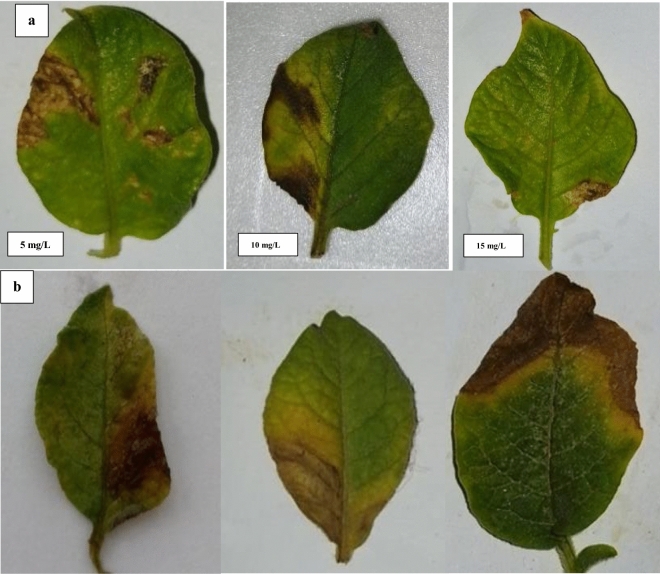
Assessment of Ferric Oxide Nanoparticles (FNPs) against *Alternaria solani* disease in detach leaf assay (**a**): Supplemented leaves, (**b**): control.

The results of the leaf-detach assay demonstrated a dose-dependent response to the application of FNPs with higher concentrations leading to a significant reduction in both disease incidence and severity caused by *A. solani* (Table 1, Fig. 7). In the leaf detach assay various concentrations of FNPs were applied to investigate their impact on disease incidence (DI) and disease severity (DS) caused by *A. solani*. The concentrations of FNPs tested were 15, 10, and 5 mg/L along with a control group that received no treatment (0). The results revealed a notable decrease in both DI and DS as the concentration of FNPs increased. In the control group (0 concentration) the DI was substantially higher at 97.33% indicating a high incidence of the disease. As the concentration of FNPs increased to 15, 10, and 5 mg/L there was a consistent reduction in DI with the lowest value recorded at 34.43% for 5 mg/L concentration. This suggests that the application of FNPs contributed to a significant reduction in the occurrence of the disease. Similarly, the DS exhibited a similar trend showing a considerable decrease with higher concentrations of FNPs. The control group had the highest DS at 34.43% indicating severe disease severity. However, as the concentration of FNPs increased, there was a consistent reduction in DS with the lowest value recorded at 27.57% for the 5 mg/L concentration. This indicates that the application of FNPs not only reduced the incidence of the disease but also mitigated its severity^27,37^.

**Table 1:** Assessment of ferric oxide nanoparticles (FNPs) against *Alternaria solani* disease in detached leaf assay.

Treatment	Concentrations (mg/L)	DI%	DS%
FNPs	15	48.57%	13.32%
	10	64.31%	18.65%
	5	73.18%	27.57%
Control	0	97.33%	34.43%

### Green house assay

**Figure 8:**
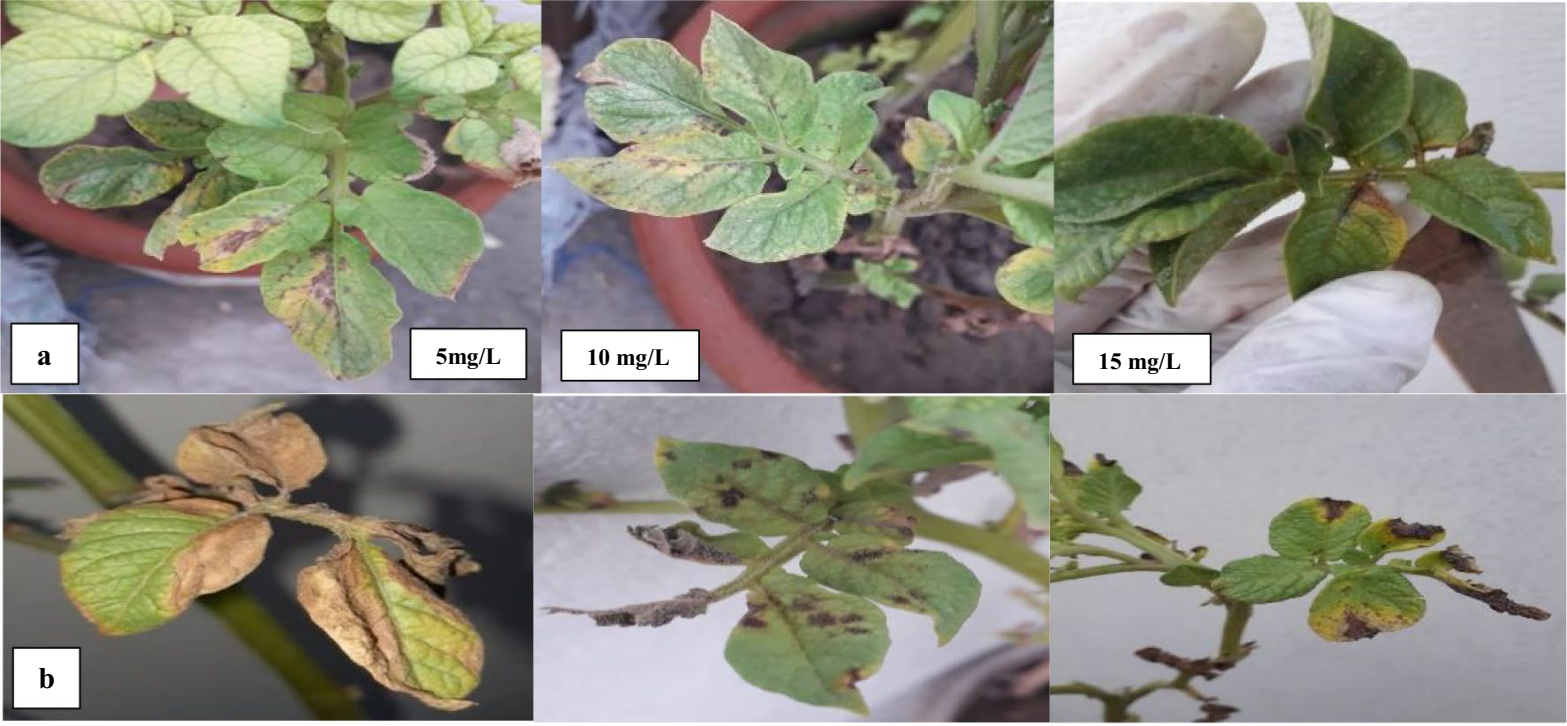
Assessment of FNPs against *Alternaria solani* disease in Greenhouse Assay (**a**): Treated groups (**b**) control groups.

In the Greenhouse Assay, the effectiveness of FNPs against *A. solani* infection was assessed through a series of concentrations. The treatments involved varying concentrations of FNPs with concentrations of 15, 10 and 5 mg/L being compared to a control group that received no FNP treatment (Table 2, Fig. 8). In the control group with no FNP treatment, the DI values were 13.33%, 23.44% and 32.33% for concentrations of 15, 10 and 5 mg/L respectively. Correspondingly, the DS values for the control group were 8.22%, 15.85% and 18.57% at the same concentrations. These results suggest that *A. solani* disease incidence and severity were observed in the absence of FNP treatment. In contrast, as the concentration of FNPs increased a notable decrease in DI and DS was observed. At the highest concentration of 15, the DI dropped to 41.00% and the DS decreased to 24.87%. This indicates a significant reduction in the incidence and severity of *A. solani* disease with the application of higher concentrations of FNPs. The concentration-dependent response highlights the potential efficacy of FNPs in mitigating the impact of the fungal pathogen in greenhouse conditions. These findings highlight the potential of FNPs as a biocontrol agent against *A. solani* demonstrating a concentration-dependent effect on disease incidence and severity. The results suggest further investigations into the optimal concentration and application methods of FNPs for enhanced plant immunity in agricultural settings^38^.

**Table 2:** Assessment of FNPs against *Alternaria solani* disease in Greenhouse assay.

Treatment	Concentration (mg/L)	DI%	DS%
FNPs	15	13.33%	8.22%
	10	23.44%	15.85%
	5	32.33%	18.57%
Control	0	41.00%	24.87%

Iron is crucial for various metabolic activities in plants, and its deficiency can impede root development. It plays a vital role in the initiation of photosynthesis and chlorophyll synthesis. Iron Oxide nanoparticles contribute to enhanced plant growth and development by dispersing throughout different plant parts and stimulating growth-promoting compounds^39^. Application of FNPs to selected groups showed no adverse effects on overall plant development. Previous studies have highlighted the potential of Iron Oxide nanoparticles in promoting plant growth, enhancing stress resistance, and serving as nutrient suppliers^40,41^. These nanoparticles have demonstrated an affinity for contaminants like arsenic. Early blight disease caused by *A. solani* poses a threat to tomato cultivation and its severe cases can lead to deterioration in stems and rarely in fruits^42^. This could be attributed to the natural defense mechanisms that plants deploy against fungal infections. Additionally, the use of nanoparticles has shown success in controlling plant diseases^43^.

Iron is essential for chlorophyll synthesis, impacting potato yield^44^. Agronomic biofortification achieved through various application methods including foliar and soil application is crucial for enhancing iron content in potatoes. The study emphasizes the importance of effective iron fertilizer types and optimal fertilizer management techniques for successful biofortification^45,46^. In the context of plant protection especially for potato plants, the study highlights the potential of nanotechnology in contemporary plant security methods. Pre-planting dressing of potato tubers with nano preparations is seen as a promising approach to boost yields, enhance product quality, reduce herbicide use, and protect against pests. The study also shows the impact of metal nanoparticles on soil biocenosis and emphasizes the need for further investigation into nanoparticles’ ecotoxicity^47^.

## Conclusion

This study introduces an eco-friendly approach for synthesizing Ferric Oxide nanoparticles (FNPs) using *Psidium guajava* leaves. The successful synthesis and structural attributes of the nanoparticles were confirmed through various characterization techniques including FTIR, UV–Vis spectroscopy, SEM, EDS and XRD. The nanoparticles exhibited a fine crystalline structure and demonstrated potential applications in disease resistance, notably reducing the severity and incidence of *Alternaria solani* in potato crop. Furthermore, the study explored the agronomic benefits of Ferric Oxide nanoparticles highlighting their role as a micronutrient in biofortification contributing to enhanced plant growth and development. This green synthesis method opens avenues for sustainable and effective nanoparticle production with potential applications in agriculture and crop protection.

## Data Availability

The original data is presented in the article. There is no supplementary data.
